# Sexual Dysfunction and Satisfaction in Males With Alcohol Dependence: A Clinic-Based Study From Central India

**DOI:** 10.7759/cureus.17492

**Published:** 2021-08-27

**Authors:** Sucharita Mandal, Sangha Mitra Godi, Mamidipalli Spoorthy

**Affiliations:** 1 Psychiatry, All India Institute of Medical Sciences, Kalyani, Kalyani, IND; 2 Psychiatry, All India Institute of Medical Sciences, Raipur, Raipur, IND; 3 Psychiatry and Behavioral Sciences, Jawaharlal Nehru Medical College, Wardha, IND

**Keywords:** alcohol dependence, sexual dysfunction, satisfaction, quality of life, asex

## Abstract

Background

Apart from the alcohol effects on various domains of health, the effect on sexual health is the most concerning aspect to the individual. Chronic alcohol intake leads to sexual dysfunction leading to interpersonal difficulties which further worsens alcohol dependence creating a vicious cycle.

Methodology

This is a cross-sectional study done at an inpatient psychiatry ward of a tertiary care hospital after taking institutional ethical clearance and due informed consent from the participants. The study sample comprised of 50 alcohol dependent subjects and 50 healthy controls taken by purposive sampling based on the inclusion criteria. Subjects were rated on the Arizona sexual experiences (ASEX) scale for various aspects of sexuality and on the New sexual satisfaction scale (NSS) for the degree of sexual satisfaction. WHO-Quality of Life (WHOQOL)-BREF was used to assess the quality of life in both groups. Data was collected and analyzed using MS Excel and SPSS version 23 (IBM Corp., Armonk, USA),

Results

The prevalence of sexual dysfunction in the study was about 40% with an inability to reach and satisfaction with orgasm (38% and 28% respectively) the most common followed by erectile dysfunction (26%). The patients with alcohol dependence had a significantly higher degree of sexual dysfunction, poor sexual satisfaction, and low quality of life compared to controls. With correlation analysis, the total scores on ASEX were positively correlated with the duration of alcohol use and dependence.

Conclusions

This study concludes that sexual dysfunction is common and seen in nearly half of the patients with alcohol dependence affecting desire, erection, and satisfaction with orgasm. Alcohol dependence further impairs the sexual satisfaction and quality of life of the individual. This information can be utilized in motivational interviewing of patients with alcohol dependence by addressing both the problems simultaneously to improve sexual functioning and quality of life.

## Introduction

According to WHO report 2014, 30% of the total population consumed alcohol in a year, of which 50% fell under the category of hazardous drinking [1]. The average age of initiation of alcohol use is also been reduced from 28 years during the 1980s to 17 years in 2007 [2]. Reports are showing an increasing pattern of alcohol dependence in India. In a report published in 2015, per capita consumption of alcohol in India has increased by 55% [3]. A recent report from the National Mental Health Survey (2015-16) in India showed that there is a high prevalence of substance use disorders (SUDs) of 22.4%, of which the prevalence of alcohol use disorder (dependence and harmful use) was 4.6%. The report states that India has been rated 4 on a scale of 0-5 in the “Years of Life Lost Scale” [4]. The report of the National Survey on Extent and Pattern of Substance Use in India, 2019 showed that Chhattisgarh is one among the states with a higher prevalence of alcohol use disorders (>10%) [5]. Alcohol use affects various spheres of an individual life like physical health, mental health, sexual health, social life, and financial aspects. It has various deleterious effects on the health of an individual leading to sexual dysfunction and interpersonal difficulties which further worsens alcohol dependence. Contrary to the past notion of alcohol being an aphrodisiac, chronic long-term use can cause sexual dysfunction. Sexual dysfunction may range from reduced sexual desire to difficulty achieving an erection, delayed orgasm, and premature ejaculation [6].

There are various mechanisms that have been postulated to explain the relationship between alcohol and sexual dysfunction. One of the mechanisms is by altering hypothalamic-pituitary-adrenal and the hypothalamic-pituitary-gonadal axis where there is inhibition of hypothalamic gonadotropin-releasing hormone and/or pituitary luteinizing hormone, thereby reduction in plasma testosterone levels [7,8]. Another mechanism is through action on amino acid receptors, increasing the inhibitory activity of gamma-aminobutyric acid receptor and decreasing the excitatory activity of glutamate receptor in the central nervous system (CNS). Ethanol also impairs spinal reflex which causes decreased sensation and decreased innervation for erection, which is speculated as a cause in erectile dysfunction with alcohol use. The psychological factors such as lack of arousability and disinterest in sex among partners - due to aversion, rejection, retaliation for her husband's undesirable drinking behavior, and psychiatric comorbidities such as anxiety and depression as well as psychotropic medications were also implicated in the sexual dysfunction secondary to alcohol use [9]. A cross-cultural study for alcohol and high-risk sexual behavior across eight countries reported that 12% of males in the general population consumed alcohol prior to first sexual intercourse due to the perceived positive effect of alcohol to improve sexual pleasure. Alcohol in small quantity in healthy individuals has been seen to be associated with enhanced sexual receptivity in women and facilitate arousal to erotic stimuli in men. Sexual dysfunction was found in 61% of patients of alcohol-dependent patients, the most common being erectile dysfunction [10,11]. Only a few Indian studies assessed the magnitude of sexual dysfunction in alcohol-dependent patients. The use of standardized instruments to evaluate sexual dysfunction was not done commonly. The studies have shown that the prevalence of sexual dysfunction ranged from 37-72%, with erectile dysfunction, reduced desire being the common areas affected [6,12-14]. The predictors of sexual dysfunction in alcohol-dependent subjects as per these studies were duration, the severity of dependence, amount of alcohol consumed. Few studies also have shown that these patients have impaired sexual satisfaction [6,12-15]. In view of the deleterious effects of alcohol on human sexuality, this study is undertaken to assess the prevalence of sexual disorders in patients with alcohol dependence and the degree of sexual satisfaction in central India.

## Materials and methods

Objectives

· To study the prevalence of sexual dysfunction among alcohol-dependent patients admitted to the psychiatry ward

· To assess the degree of sexual satisfaction in alcohol-dependent patients during the past six months and compare it with healthy controls

· To assess the quality of life of the alcohol-dependent patients and compare it with healthy controls

Materials and methods

The current study was a cross-sectional survey conducted at a tertiary care hospital. Institutional ethics committee clearance was taken prior to conducting the study. It was carried out over a period of 1 year (June 2018-June 2019).

Participants

It included two groups - a study group and a control group - recruited through purposive sampling. Written informed consent was taken from all the study participants prior to inclusion and assessment. Fifty patients with the diagnosis of alcohol dependence syndrome as per the International Classification of Diseases (ICD-10) Diagnostic Criteria for Research (DCR) attending the psychiatry outpatient/inpatient settings are included in the study group. Fifty healthy controls were recruited from the department as per inclusion criteria.

We have included males aged between 18-60 years, who were married or had been sexually active, able to read and understand Hindi in both the groups. Patients who were in acute alcohol withdrawal or intoxication, who had substance abuse other than caffeine, alcohol were excluded from the study group. Those with medical and surgical illnesses which can cause sexual dysfunction, like hypertension, diabetes mellitus, thyroid dysfunction, cardiovascular disorders, renal dysfunctions, and neurological disorders, intellectual disability using medication affecting sexual functioning (assessed by history, examination, previous records/investigations) were excluded from both the groups. Apart from these criteria, subjects with any psychiatric comorbidity, substance use apart from tobacco and caffeine were excluded from the control group.

Research Tools

Following assessments were carried out in the study population

1. Demographic information of both study and control groups was recorded using the standard format of socio-demographic profile.

2. General Health Questionnaire (GHQ-5) was used to ascertain the selection criteria in control groups. A shorter, five-item version of the GHQ was used to screen healthy controls. It is simple, easy to administer, and has acceptable sensitivity and specificity. It is also validated for use in the Indian population [16].

3. Clinical Institute Withdrawal Assessment of Alcohol Scale, Revised (CIWA-Ar) was applied on patients prior to inclusion in the study group. It was used to screen for withdrawal state. Patients who scored < 10 and were not in acute withdrawal were included. It is a clinician-rated scale and takes five minutes to administer [17].

4. Subjects were rated on Arizona Sexual Experiences Scale (ASEX) to assess the various aspects of sexuality. It is a five-item self-report inventory using a six-point Likert scale method. It evaluates sexual function in men and women, regardless of sexual orientation or relationship with a partner. Each item from the scale measures one domain of sexual function like drive, arousal, penile erection/vaginal lubrication, ability to reach orgasm, and satisfaction from orgasm. Clinical sexual dysfunction is measured by this scale using the following criteria: a total score > 19 on ASEX or a score > 5 on any one item or a score > 4 on any three items. Higher global scores include greater sexual dysfunction. The reliability and validity of the scale were found to be excellent. A validated Hindi version of the scale was used for the current study [18].

5. New sexual satisfaction scale was used to assess the degree of sexual satisfaction in the past six months. It is a self-rated scale that covers various aspects of sexual satisfaction like sexual sensations, sexual awareness and focus, sexual exchange, emotional closeness, and sexual activity. It has 20 items, each item measured on a Likert scale of 1-5. Higher scores indicate greater satisfaction. It has satisfactory reliability and validity and can be used irrespective of the subject’s gender, sexual orientation, and relationship status. A validated Hindi version of the scale was used for the purpose of the study [19].

6. WHO-Quality of Life (WHOQOL)-BREF was used to assess the quality of life in both groups. This is a standardized instrument that is translated into the Hindi language. WHOQOL-BREF version assesses the quality of life under four subsections: physical health, psychological health, social relationships, environment. Each item is rated on a score ranging from 1-5 [20].

Data Analysis

Data analysis was performed using SPSS Statistics software (version 23, IBM Corp., Armonk, USA) and Microsoft Excel worksheet 2016 (Microsoft Corporation, Redmond, USA). Descriptive analysis is used to describe the data and distribution of variables quantitatively. The association between the variables was analyzed using an independent t-test for continuous variables and a Chi-square test for categorical variables. A correlational analysis is used to evaluate the association between sets of continuous variables. A p-value less than 0.05 is considered statistically significant at 95% confidence intervals.

## Results

Table 1 shows the sociodemographic profile of the study sample. The mean age of cases and controls was about 38 years and 35 years, respectively; no significant difference was found in the age and marital status of both the groups. However, in our sample, controls had a significantly higher duration of education compared to the alcohol-dependent subjects. The mean duration of alcohol consumption in the subjects with alcohol dependence is almost double the mean duration of alcohol dependence.

**Table 1:** Socio-demographic details of cases and controls ** Highly significant level with p value less than 0.001

Variable	Case (%) (Mean ± SD)	Control (%) (Mean ± SD)	Chi-square/t-test (p)
Age (in years)	38.64 ± 9.65	35.90 ± 10.23	1.37(0.172)
Married	92	82	2.21(0.137)
Single	8	18	
Education (in years)	11.62 ± 4.32	14.38 ± 3.50	3.50 (0.001)**
Education			
Illiterate	2	2	-
Primary School	10	2	
Middle School	20	6	
High school	28	18	
Intermediate	18	32	
Graduate/Postgraduate	22	40	
Occupation			
Unskilled worker	16	6	-
Semiskilled worker	22	14	
Skilled worker	10	30	
Clerical/shop owner/farmer	26	20	
Semi Professional	16	22	
Professional	10	8	
Religion			
Hindu	94	88	-
Muslim	2	10	
Christian	4	2	
Alcohol use in years	12.98 ± 7.99	N/A	-
Alcohol dependence in years	5.92 ± 4.96	N/A	-

Table 2 depicts the pattern of sexual dysfunction and different domains of sexual dysfunction as per the Arizona Sexual Experiences Scale (ASEX). Sexual dysfunction was defined as per ASEX as a global score ≥ 19 or a score of 4 in at least three domains or a score of 5 in any one domain. The prevalence of sexual dysfunction as per the above definition of ASEX is about 40% in the alcohol-dependent subjects. A cut-off score of 4 was used to define sexual dysfunction among individual domains. The most common form of sexual dysfunction was the inability to reach orgasm seen in 38% of the alcohol-dependent subjects, followed by dissatisfaction with orgasm (28%), problems in erection (26%); difficulty in arousal (24%) and problems in desire were least affected, accounting for 24%.

**Table 2:** Prevalence rate of sexual dysfunction as per ASEX ASEX: Arizona Sexual Experiences Scale

Variables	Alcohol-dependent subjects (n=50)
ASEX global score ≥ 19	12 (24%)
ASEX score 4 on three domains but global score < 19 or score 5 on one domain but global score < 19	8 (16%)
Total number of patients with sexual dysfunction	20 (40%)

Table 3 shows the patients with alcohol dependence had a significantly higher degree of sexual dysfunction scores as measured by ASEX compared to the control group. Also, there was a significant difference in four out of five domains of ASEX, like dysfunction in desire, erection, ability to reach orgasm, and satisfaction with orgasm, among alcohol-dependent individuals. As per New Sexual Satisfaction Scale (NSSS), sexual satisfaction was found to be poor in alcohol-dependent cases compared to controls with a significant difference between both the groups. The quality of life as measured on WHOQOL-BREF version showed that patients with alcohol dependence had a significantly poor quality of life compared to healthy controls (Table 4).

**Table 3:** Sexual dysfunction as assessed by domains of ASEX ASEX: Arizona Sexual Experiences Scale * Significant level with p value less than 0.05 ** Highly significant level with p value less than 0.001

Domains of ASEX	Cases (⸓)	Controls (⸓)	Chi-square test (p-value)
Impaired sexual desire			
Yes	24	4	8.306 (0.004)*
No	76	96	
Impaired arousal			
Yes	24	6	6.353 (0.012)*
No	76	94	
Impaired erection			
Yes	26	0	14.94(<0.001)**
No	74	100	
Impaired ability to achieve orgasm			
Yes	38	2	20.25(<0.001)**
No	62	98	
Impaired satisfaction with the orgasm			
Yes	28	8	6.77(0.009)*
No	72	92	

**Table 4:** Sexual dysfunction, satisfaction, and quality of life ASEX: Arizona Sexual Experiences Scale; NSSS: New Sexual Satisfaction Scale * Significant level with p value less than 0.05 ** Highly significant level with p value less than 0.001

Variable	Case (Mean ± SD)	Control (Mean ± SD)	t-test (p value)
NSSS total	67.66 ± 23.90	79.40 ± 18.82	2.72(0.008)*
ASEX total	15.02 ± 4.67	11.94 ± 3.06	3.89(<0.001)**
Desire	2.94 ± 0.99	2.46 ± 0.76	2.70(0.008)*
Arousal	2.82 ± 1.17	2.50 ± 1.01	1.45(0.148)
Erection	3.04 ± 1.26	2.24 ± 0.65	3.97(<0.001)**
Orgasm	3.22 ± 1.34	2.36 ± 0.80	3.88(<0.001)**
Satisfaction	3.00 ± 1.34	2.38 ± 0.83	2.78(0.007)*
WHOQOL-BREF	82.54 ± 16.92	89.24 ± 15.28	2.07(0.04)*

Correlation analysis has showed that there is a significant positive correlation between the age of the individual and alcohol dependence with higher the age, more the years of alcohol use and dependence. However, there was a negative correlation between education and alcohol dependence, with lower the duration of education in years, higher the duration of alcohol use and dependence. The study also found that the total scores on ASEX were positively correlated with the duration of alcohol use and dependence, whereas the scores on the NSSS scale and WHOQOL-BREF were negatively correlated with the duration of use and duration of alcohol dependence. But the correlations were not statistically significant (Table 5).

**Table 5:** Results of correlation analyses ASEX: Arizona Sexual Experiences Scale; NSSS: New Sexual Satisfaction Scale * Significant level with p value less than 0.05 ** Highly significant level with p value less than 0.001

Variables	Alcohol use in years (Pearson’s r/p)	Alcohol dependence in years (Pearson’s r/p)
Age	0.605(<0.001)**	.560(<0.001)**
Education in years	-0.237(0.151)	-.301(0.066)
ASEX total scores	0.214(0.197)	.814(0.270)
NSSS total scores	-.061(0.714)	-.070(0.676)
WHOQOL-BREF Total Scores	-.108 (0.517)	-.057(0.734)

## Discussion

The current study tried to assess the relationship between alcohol dependence and sexual dysfunction, sexual satisfaction, impact on the quality of life of these individuals in comparison with healthy volunteers. The mean age of the alcohol-dependent subjects and controls in the present study is about 39 years and 36 years, respectively, with no significant difference which removes the age bias while comparing the groups. This finding is in concordance with the previous studies done in India [6,14,15]. It was found that healthy volunteers had a significantly higher duration of education compared to alcohol-dependent subjects; in patients, the lower the duration of education in years, the higher was the duration of alcohol use and dependence. This implies that poor literacy status makes individuals unaware of the costs of alcohol consumption and deleterious effects of alcohol on health and different spheres of their lives. This is in line with the studies done by Pendharkar et al and Dissiz and Oskay 2011 [13,21]. However, contrasting findings were shown by Saha, 2017 where no significant difference was found regarding educational status [14]. The mean duration of alcohol consumption in the subjects with alcohol dependence is 12.98 ± 7.99 years with a mean duration of alcohol dependence of about 5.92 years which is in line with the study by Anil Kumar et al 2016 [15]. Our study had found that the prevalence of sexual dysfunction as per the definition of ASEX is about 40% in the alcohol-dependent subjects which is slightly lower than the prevalence rates in other studies by Pendharkar et al and 2016 Anil Kumar et al, 2016 [13,15].

Data from Indian settings suggest that the prevalence rates of sexual dysfunction among alcohol-dependent patients vary widely. This difference in the reported rates in sexual dysfunction might be due to the shame and stigma attached with disclosure of sexual problems particularly in countries like India. The most common type of sexual dysfunction in the present study was the inability to reach orgasm, followed by erectile dysfunction. This is in line with the study by Anil Kumar et al 2016 which also found that anorgasmia is the most common sexual dysfunction followed by erectile dysfunction [15]. Contrasting with this finding of ours, most of the studies reported erectile dysfunction as the most common sexual disorder in alcohol-dependent subjects, followed by poor sexual desire. This might be due to the variation in the scales used for assessment of sexual dysfunction apart from the reason that erectile dysfunction can be easily appreciated compared to anorgasmia, the concept of which is unknown to patients.

In our study, patients with alcohol dependence had greater sexual dysfunction in almost all areas like impaired desire, erection, orgasm compared to healthy individuals. These findings are consistent with the results of the previous literature [12,13,22]. However, the study by Schiavi et al, 1995 found that there was no difference between alcoholic subjects and non-alcoholic controls in any domain of sexual function or the prevalence of sexual problems [23]. This might be due to the small sample size of the study.

As per New Sexual Satisfaction Scale (NSSS), sexual satisfaction was found to be significantly poor in alcohol-dependent cases whereas the sexual satisfaction scores were higher in healthy volunteers. Similarly, studies by Saha, 2017 and O’Farrell et al, 1997 also found that alcohol dependence was associated with poor overall sexual satisfaction as assessed by the Brief Sexual Function Inventory (BSFI), International Index of Erectile Function (IIEF), and Index of Premature Ejaculation (IPE) [14,24]. The quality of life as measured on WHOQOL-BREF version showed that patients with alcohol dependence had a significantly poor quality of life compared to healthy controls. The quality of life in alcohol-dependent individuals also decreases with an increase in years of alcohol use and dependence and this finding is consistent with previous studies [25,26]. The current study findings have shown that duration of alcohol use and alcohol dependence are positively correlated with scores on ASEX. This implies that with an increase in years of alcohol use and dependence, the sexual dysfunction increases. These findings were consistent with the study by Saha [14] however in contrast to study by Arackal and Benegal [12] where no association was found between the duration of alcohol use and sexual dysfunction. The duration of alcohol use and alcohol dependence were negatively correlated with scores on NSSS indicating as the years of alcohol use and dependence increase, the sexual satisfaction decreases.

Our study is not without limitations. The small sample size of the study in both cases and controls limits the generalizability of the findings. Some aspects affecting sexual dysfunction like marital discord have not been studied, which might have lead to inflated rates of sexual dysfunction. Certain predictors of sexual dysfunction in alcohol dependence patients like the severity of alcohol dependence and the amount of alcohol used were not assessed in the current study. Matching of both the groups was not done and the confounding effect of tobacco could not be assessed.

## Conclusions

We found that sexual dysfunction is common in male patients with alcohol dependence. It is found from our study that sexual satisfaction is also impaired in alcohol-dependent individuals, which in turn hampers the quality of life of these individuals. This might create a vicious cycle in which the individuals seek more alcohol intake for more satisfaction in their lives but the problem remains. This information can be utilized in motivational interviewing of patients with alcohol dependence syndrome. So, psychoeducation of individuals on this aspect might also reduce the burden of alcohol intake. Clinicians dealing with alcohol-dependent patients must be knowledgeable about the associated sexual dysfunction and should address both the problems simultaneously to improve their functioning and quality of life. Such studies should be carried out on a larger scale, especially from Central India, where the focus of research on this aspect is lacking.
